# Insufficient Ablation is Associated with Atrial Fibrillation Recurrence after Combining Ablation and Left Atrial Appendage Closure

**DOI:** 10.31083/j.rcm2501010

**Published:** 2024-01-09

**Authors:** Xueyan Ding, Yao Zhao, Shaohua Dong, Xinmiao Huang, Aihong Qin, Jiang Cao, Zhifu Guo, Songqun Huang

**Affiliations:** ^1^Department of Cardiology, Sir Run Run Shaw Hospital, School of Medicine, Zhejiang University, 310016 Hangzhou, Zhejiang, China; ^2^Department of Cardiovasology, Changhai Hospital, Second Military Medical University, 200433 Shanghai, China

**Keywords:** atrial fibrillation, hybrid procedure, left atrial appendage closure, catheter ablation, insufficient ablation

## Abstract

**Background:**

The combination of left atrial appendage closure (LAAC) and catheter ablation (CA) in a single procedure is a safe and effective form of treatment for atrial fibrillation (AF). However, several findings have argued that LAAC might increase the risk of AF recurring. Therefore, this study investigated the impact of insufficient ablation on AF recurrence after the hybrid procedures of CA and LAAC.

**Methods:**

We reviewed 107 consecutive patients with AF who received the CA and LAAC hybrid procedures (combined group). In the case–control study, another 107 patients who underwent only CA (ablation group) were successfully matched using propensity score matching. After correcting the insufficient ablation, 107 consecutive patients were enrolled prospectively. During the follow-up period, postprocedural 24-hour monitor recordings and a portable electrocardiogram (ECG) monitoring device were used to detect AF recurrence. Transesophageal echocardiography was used to evaluate LAAC.

**Results:**

The combined group showed an increase in the risk of AF recurrence after 539.2 ± 304.4 days of follow-up (29.9% vs. 15.9%, *p* < 0.05). Interestingly, the duration of the procedure was not significantly prolonged when LAAC was added after CA in the combined group, while there was a higher number of ablating attempts, duration of ablation, and additional ablation in the ablation group for both radiofrequency and cryoballoon ablation. After correcting for the insufficient ablation, the corrected group showed a significant decrease in AF recurrence after 420.4 ± 204.8 days of follow-up.

**Conclusions:**

Insufficient ablation is common when combining CA and LAAC and may lead to the recurrence of atrial fibrillation. It should be corrected intentionally by sufficient ablation of the pulmonary vein antrum and additional ablation.

**Clinical Trial Registration:**

The prospective study is a sub-study of our CAGEDAF study that has already been registered (ChiCTR2000039746).

## 1. Introduction

Atrial fibrillation (AF) is responsible for an increased risk of thromboembolic stroke and impaired quality of life [1]. Catheter ablation (CA), including radiofrequency ablation and cryoballoon ablation, has become a standard procedure to attenuate the symptoms and improve the quality of life of patients with AF, by reducing the AF burden [2]. However, previous studies have failed to find significant reductions in the risk of stroke after AF ablation [3, 4]. Consequently, clinical guidelines recommend antithrombotic therapy in AF patients to reduce the risk of stroke even after catheter ablation [5]. Additionally, the issue of bleeding risk in patients with continuous anticoagulation after AF ablation has received considerable critical attention [6].

In an effort to reduce both the risk of embolism and bleeding, left atrial appendage closure (LAAC) has been widely used in patients with a high ${\mathrm{CHA}}_{2}$${\mathrm{DS}}_{2}$-VASc score and a high HAS-BLED score. Therefore, for this particular population, the hybrid procedures of LAAC and CA have become an alternative treatment strategy [7]. The greater part of the research on the hybrid procedures of CA and LAAC has emphasized the safety and feasibility of this hybrid regimen since they can not only be used to improve symptoms and quality of life but also to significantly reduce the risk of stroke [8, 9]. In contrast, there are also some other studies that argue that left atrial structural remodeling after LAAC may lead to a higher risk of AF recurrence [10, 11]. However, these previous findings remain inconclusive. Therefore, a case–control study was performed that focused on the risk factors of an increased recurrence of atrial tachycardia or AF after the hybrid procedure. Successively, a prospective study was conducted on improving the efficiency of AF ablation after correcting for insufficient ablation.

## 2. Materials and Methods

### 2.1 Case–Control Study

Between January 2017 and December 2018, there were 107 symptomatic nonvalvular AF patients (combined group) included in this single-center study, who underwent CA combined with LAAC at Changhai Hospital. The inclusion criteria were a HAS-BLED score ≥3 or intolerance to chronic oral anticoagulation. The exclusion criteria were as follows: (1) aged ≤18 years; (2) LA thrombus; (3) LA diameter ≥55 mm. Controls were selected by propensity score matched analysis, using age, sex, HAS-BLED score, and ${\mathrm{CHA}}_{2}$${\mathrm{DS}}_{2}$-VASc score to reduce potential confounding bias. Patients in the ablation group were matched 1:1 with patients in the combined group (**Supplementary Material**). AF-free survival rate was compared between the combined group and the ablation group.

### 2.2 Radiofrequency Ablation Procedure

Radiofrequency ablation procedure was performed with an uninterrupted direct oral anticoagulant. Activated clotting time (ACT) was targeted between 300 and 350 seconds throughout the procedure. A coronary sinus catheter was positioned in the coronary sinus (CS) through the left femoral vein and through a right ventricle catheter in the right ventricle. Following transseptal puncture, point-by-point ablation was performed using a Tacticath Quartz catheter (Abbott Inc, St. Paul, MN, USA) and power-control mode with a power of 30–40 W within the LA. All patients underwent pulmonary vein isolation (PVI). According to the discretion of the operator, patients underwent different additional ablation strategies, including roof, mitral linear ablation, etc.

### 2.3 Cryoballoon Ablation Procedure

Following transseptal puncture, a 28 mm cryoballoon (Arctic Front Advance, Medtronic, MN, USA) was inserted into the left atrium. After insertion, the cryoballoon was inflated and wedged into the ostia of the pulmonary veins, and contrast medium was injected to confirm perfect pulmonary vein (PV) occlusion. A 180-second freeze was delivered when the time to PVI was <60 seconds. Achieve catheter should be positioned to avoid it being too far from the distal portion of the cryoballoon, to make the pulmonary vein activity recording simpler. Attempts were also made to determine the accurate time to PVI. To avoid phrenic nerve palsy, the operator paced the ipsilateral phrenic nerve at maximum output (12 V at 2.9 ms) with a cycle length of 1000 ms during cryogenic ablation of the right pulmonary vein. The activated clotting time was the same as the radiofrequency ablation procedure. Pulmonary vein antrum ablation and additional ablation strategies for each case were at the discretion of the primary operator.

### 2.4 LAAC Procedure

Following the ablation procedure, the Watchman device (Boston Scientific, Marlborough, MA, USA) was implanted. The previously used FlexCath sheath or Agilis NxT sheath was replaced by a 14F sheath. Under the protection of the pigtail catheter, the sheath for the Watchman device was delivered to the ostium of the left atrial appendage (LAA). Angiography of the LAA was performed from multiple angles and based on that the proper Watchman device was selected and released into the correct position. Before releasing the device, angiography was performed again to ensure that the position of the device was correct and that no- or minimal residual blood flow was observed.

### 2.5 Prospective Study

**Fig. 1.**
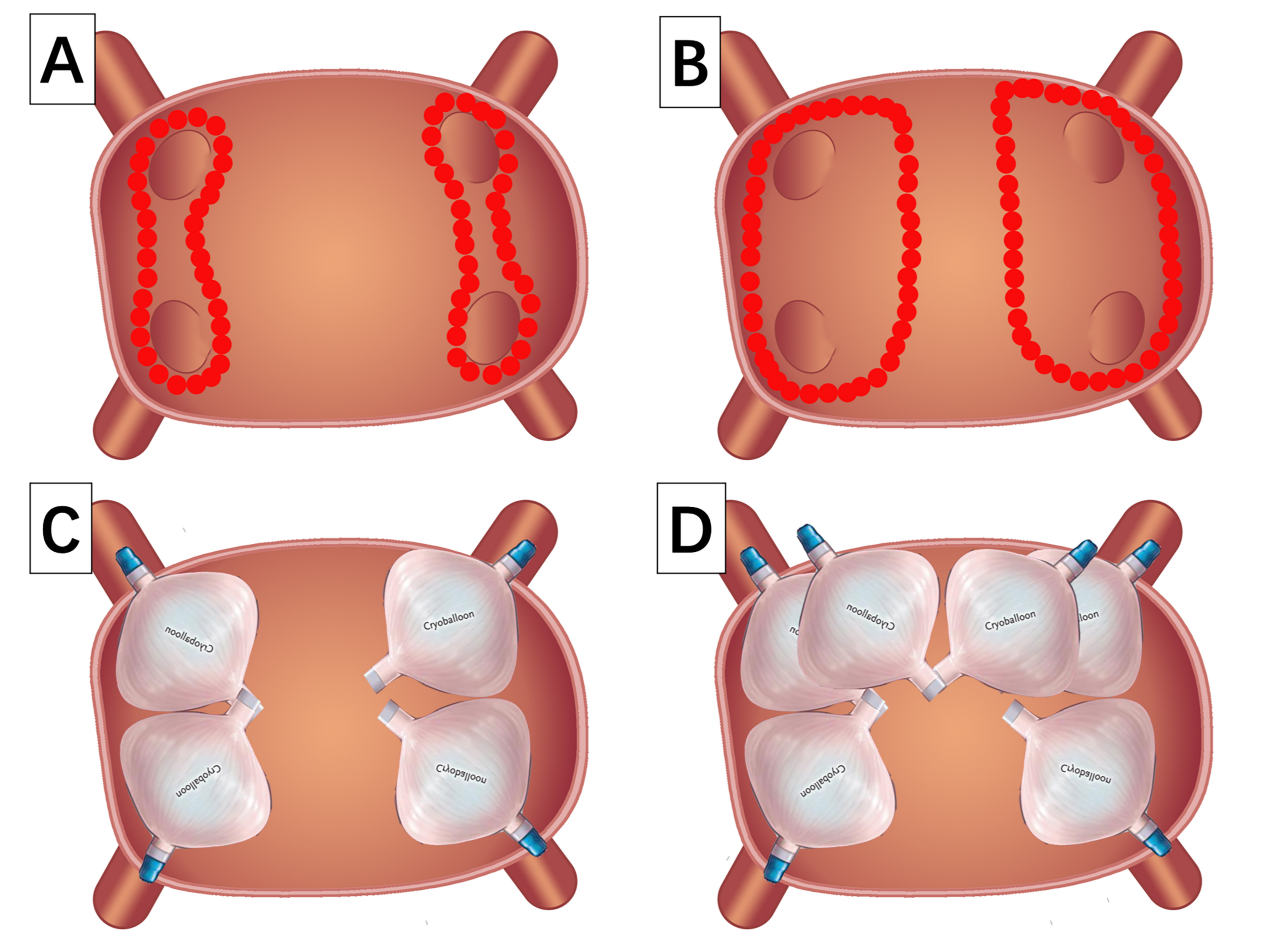
**Comparison of insufficient ablation and corrected ablation**. (A) Insufficient ablation of radiofrequency ablation for pulmonary vein antrum. (B) Corrected ablation of radiofrequency ablation for pulmonary vein antrum. (C) Insufficient ablation of cryoballoon ablation for pulmonary vein antrum. (D) Corrected ablation of cryoballoon ablation for pulmonary vein antrum.

Based on the results of the case–control study, we speculated that insufficient ablation is a correctable risk factor that is associated with AF recurrence. A standard wide circumferential pulmonary vein antral isolation approach and suitable additional ablation were used to correct the insufficiency ablation in both the radiofrequency ablation and cryoballoon ablation, as shown in Fig. 1. Successively, we prospectively enrolled 107 patients undergoing AF ablation combined with LAAC on a corrected strategy (corrected group). The pulmonary vein antrum was ablated sufficiently, and a suitable additional ablation strategy was used intentionally. The AF-free survival rate of the corrected group was compared to the other two groups. The study flowchart is shown in the graphical abstract.

### 2.6 Arrhythmia Recurrence Assessment

After radiofrequency ablation or cryoballoon ablation procedure, patients in the ablation group received anticoagulation for at least three months. Data were obtained by 24-hour monitoring at the 3-, 6-, and 12-month follow-up procedures to evaluate the outcome of the AF ablation. A recurrent AF is defined as an AF or atrial tachycardia that lasts longer than 30 seconds without antiarrhythmic drugs.

### 2.7 Postprocedural Anticoagulation and Left Atrial Appendage Closure Assessment

In the combined group and corrected group, a direct oral anticoagulant was recommended for three months after procedures. Dual antiplatelet drugs were prescribed in the next 3 months followed by lifelong aspirin use, if a satisfactory transesophageal echocardiography (TEE) or computerized tomography (CT) result was confirmed at the 3-month follow-up. The definition of a successful LAAC included the desired positioning of the Watchman device, complete coverage of the LAA ostium, and a peri-device flow of <5 mm. Oral anticoagulant use was recommended when the peri-device flow was >5 mm.

### 2.8 Statistical Analysis

A *t*-test was used to compare continuous variables and χ^2^ test for categorical variables. To examine differences in AF recurrence among the three groups, Kaplan–Meier survival curves and log-rank analyses were used. For survival data, univariate and multivariate Cox regression analyses were performed. The significance level for all *p* values was set at <0.05. All statistics were analyzed using SPSS (version 24, IBM, Armonk, NY, USA).

## 3. Results

### 3.1 Baseline Characteristics and Ablation Parameters in the Case–Control Study

In total, 214 patients were included in the case–control study, 107 patients were in the combined group and 107 matched controls were in the ablation group. There was no significant difference in the demographic characteristics between the two groups. Interestingly, the procedure time was not significantly increased, while the fluoroscopy time was prolonged in the combined group compared with the ablation group. There is a greater number of ablating attempts and a longer duration of ablation in the ablation group for both the radiofrequency and cryoballoon ablations. The characteristics and ablation parameters for the two groups are shown in Table 1. These results suggested that insufficient ablation may contribute to a higher recurrence of AF and atrial tachycardia in the combined group.

**Table 1.** **Baseline characteristics and ablation parameters**.

Variables		Combined ${\mathrm{group}}^{*}$	Ablation ${\mathrm{group}}^{\#}$	Corrected ${\mathrm{group}}^{\&}$	vs.	vs.
		(N = 107)	(N = 107)	(N = 107)	*p* value	*p* value
Age		67.0 ± 8.7	66.3 ± 9.9	65.2 ± 11.9	0.599	0.206
Male		56 (52.3%)	60 (56.1%)	58 (54.2%)	0.583	0.784
Paroxysmal AF (%)		77 (72.0%)	72 (67.3%)	75 (70.1%)	0.457	0.763
BMI (${\mathrm{kg/m}}^{2}$)		24.3 ± 3.0	24.7 ± 2.6	25.0 ± 2.5	0.682	0.060
${\mathrm{CHA}}_{2}$${\mathrm{DS}}_{2}$-Vasc		3.3 ± 1.2	3.2 ± 1.1	3.0 ± 1.4	0.654	0.145
HAS-BLED		2.8 ± 0.8	2.8 ± 0.7	2.7 ± 0.9	0.292	0.229
Scr (µmol /L)		75.5 ± 17.2	78.1 ± 16.8	76.5 ± 17.9	0.763	0.693
GFR (mL/min)		87.9 ± 15.6	88.0 ± 14.7	89.7 ± 16.3	0.611	0.390
ALT (U/L)		32.2 ± 14.9	34.8 ± 18.1	33.9 ± 14.2	0.208	0.374
AST (U/L)		20.2 ± 6.2	21.0 ± 5.8	19.8 ± 7.1	0.719	0.667
Serum kalium (mmol/L)		3.9 ± 0.4	3.9 ± 0.4	3.9 ± 0.4	0.295	0.975
LAD (mm)		4.1 ± 0.5	4.1 ± 0.6	4.0 ± 0.7	0.641	0.733
LAD-after (mm)		4.0 ± 0.6	4.1 ± 0.7	4.0 ± 0.8	0.216	0.992
EF (%)		61.4 ± 4.2	61.0 ± 4.4	60.0 ± 7.7	0.497	0.090
LVDD (mm)		4.4 ± 0.3	4.6 ± 0.4	4.5 ± 0.5	0.821	0.773
IVS (mm)		1.0 ± 0.1	1.1 ± 0.1	1.0 ± 0.2	0.596	0.161
BNP (pg/mL)		163.1 ± 140.1	171.0 ± 129.6	169.9 ± 108.1	0.779	0.810
MR (mL)		2.6 ± 2.2	2.5 ± 2.0	2.3 ± 1.8	0.458	0.181
TR (mL)		2.7 ± 2.7	3.1 ± 2.4	2.7 ± 2.4	0.642	0.937
AR (mL)		0.4 ± 0.4	0.3 ± 0.6	0.5 ± 0.7	0.545	0.103
Cryoballoon ablation (%)		54 (50.5%)	60 (56.1%)	56 (52.3%)	0.411	0.784
Radiofrequency ablation		N = 53	N = 47	N = 51		
	Number of ablating attempts	47.2 ± 5.3	58.8 ± 6.8	75.5 ± 17.0	<0.001	<0.001
	Duration of ablation (s)	1695.2 ± 256.7	2099.1 ± 366.8	2396.5 ± 738.1	<0.001	<0.001
	Procedure time (min)	128.3 ± 31.9	126.1 ± 28.5	142.9 ± 33.2	0.720	0.024
	Fluoroscopy time (min)	8.6 ± 2.1	4.1 ± 1.1	10.9 ± 3.6	<0.001	<0.001
	Additional ablation (%)	7 (13.2%)	17 (36.2%)	19 (37.3%)	0.007	<0.001
Cryoballoon ablation		N = 54	N = 60	N = 56		
	Number of ablating attempts	4.8 ± 0.5	6.1 ± 1.4	6.5 ± 2.2	<0.001	<0.001
	Duration of ablation	811.1 ± 61.4	996.0 ± 168.9	1179.8 ± 334.0	<0.001	<0.001
	Procedure time (min)	93.4 ± 18.1	95.4 ± 25.6	132.5 ± 42.1	0.648	0.001
	Fluoroscopy time (min)	10.1 ± 2.6	6.0 ± 2.0	13.0 ± 5.5	<0.001	<0.001
	Additional ablation (%)	0 (0%)	5 (8.3%)	6 (10.7%)	0.030	<0.001

^*^ = Combined group; ^#^ = Ablation group; ^&^ = Corrected group; BMI, body mass index; Scr, serum creatinine; GFR, glomerular filtration rate; ALT, alanine transaminase; AST, aspartate transaminase; LAD, left atrial diameter; LAD-after, left atrial diameter after follow-up; EF, ejection fraction; LVDD, left ventricular end-diastolic dimension; IVS, interventricular septal thickness; MR, mitral regurgitation; TR, tricuspid regurgitation; AR, aortic regurgitation; AF, atrial fibrillaiton; BNP, B-type natriuretic peptide; Additional ablation included pulmonary vein antrum ablation or linear ablation following pulmonary vein isolation.

### 3.2 Primary Outcome in the Case–Control Study

**Fig. 2.**
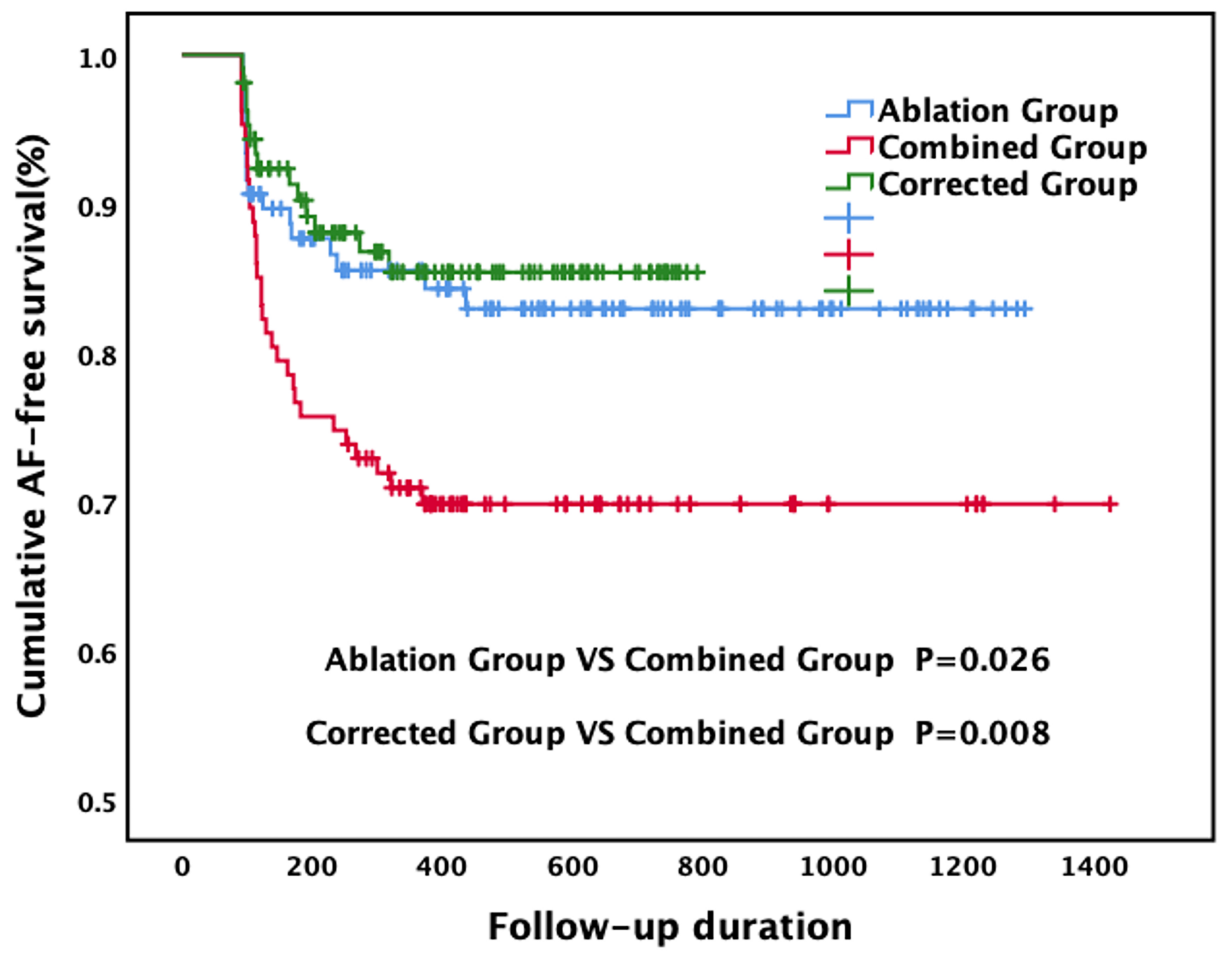
**The Kaplan–Meier survival curve for the primary endpoint**. AF, atrial fibrillation.

After 599.0 ± 340.9 days of following-up, being free from the recurrence of AF and atrial tachycardia was achieved in 90/107 (84.1%) patients (90.3% in paroxysmal AF and 71.4% in persistent AF) in the ablation group. Comparatively, in the combined group, being free from the recurrence of AF and atrial tachycardia was achieved in 75/107 (70.1%, *p* = 0.015) patients (77.9% in paroxysmal AF and 50.0% in persistent AF) after a follow-up period of 539.2 ± 304.4 days. The Kaplan–Meier curves for recurrence, stratified by groups, are shown in Fig. 2. The log-rank test revealed that recurrence of AF or atrial tachycardia was recorded significantly earlier in the combined group than in the ablation group (*p* = 0.026). A multivariate Cox model revealed that AF recurrence was associated with a larger left atrial diameter, combined group, and persistent AF (Table 2).

**Table 2.** **Cox regression analysis with AF Recurrence**.

Variable	Univariable analysis		Multivariable analysis	
	β-coefficient (95% CI)	*p*	β-coefficient (95% CI)	*p*
Groups	0.518 (0.288 to 0.934)	0.029	0.473 (0.262 to 0.852)	0.013
AF type	2.819 (1.609 to 4.939)	<0.001	2.551 (1.415 to 4.598)	0.002
LAD	1.014 (1.004 to 1.023)	0.004	1.010 (1.001 to 1.019)	0.029

LAD, left atrial diameter; AF, atrial fibrillaiton.

### 3.3 Primary Outcome in the Prospective Study

Based on the above results, we hypothesized that insufficient ablation may be associated with a higher AF recurrence when AF ablation was combined with LAAC. In the prospective study, there was no statistical difference in demographic data between patients in the combined group and the corrected group. After correcting the insufficient ablation, the corrected group showed an increase in additional ablations, number of ablating attempts, duration of ablation, procedure time, and fluoroscopy time. After a follow-up period of 420.4 ± 204.8 days, the corrected group showed an improvement in the cumulative AF-free survival rate, as shown in Fig. 2.

### 3.4 Clinical Outcome of Left Atrial Appendage Closure

Peri-device leaks of >5 mm were detected by TEE in four patients in the combined group and in five patients in the corrected group (3.7% vs. 4.7%, respectively) at the 3-month follow-up. A hemorrhagic stroke happened in one patient in the combined group, and an ischemic stroke occurred in one patient in the corrected group. Finally, one patient in the combined group died of epilepsy 6 months after the procedure.

## 4. Discussion

The main finding of our study was to demonstrate that insufficient ablation is associated with AF recurrence after hybrid procedures of ablation and LAAC. Insufficient ablation is common in CA combined with LAAC procedures that may lead to AF recurrence in a case–control study. In this prospective study, we found that AF recurrence was reduced by correcting the insufficient ablation.

Accumulating evidence has demonstrated that stroke risk in AF patients with a high ${\mathrm{CHA}}_{2}$${\mathrm{DS}}_{2}$-VASc score was not significantly reduced after AF ablation [3, 4], due to the high risk of AF recurrence or poor AF detection after CA in the population with a high stroke risk [12, 13]. Consequently, clinical guidelines recommend antithrombotic therapy in this population, even after CA. However, more recent attention has focused on the issue of bleeding risk in patients with continuous anticoagulation after AF ablation. In order to reduce both the risk of embolism and bleeding, hybrid procedures of CA and LAAC remain a sensible option for symptomatic AF patients with high risks of stroke or bleeding.

Pioneering research demonstrated that the combination of radiofrequency ablation and LAAC can be implemented safely [7]. Subsequent research expanded the sample size, extended the follow-up duration, and further confirmed the safety and feasibility of this hybrid regimen [14, 15, 16, 17, 18, 19, 20]. Recent studies have demonstrated the safety and efficacy of concomitant cryoballoon ablation and LAAC [21, 22]. The expert consensus statement by the EHRA/EAPCI [23] suggested that the hybrid procedures of CA and LAAC are an effective and practical approach due to the common aspects (e.g., transseptal puncture and anticoagulation). In contrast to the studies above, there are also some other studies that argue that the combination may lead to a higher risk of AF recurrence. Luani *et al*. [10] found that the left atrial volume increased significantly after interventional LAAC. Additional research also found significant increases in the left atrial size and decreases in the left atrial function, which may result in AF recurrence [11]. In the present study, we compared the left atrial diameters before and after the procedures in the different groups and found no significant difference in the left atrial size. Therefore, it seems likely that other factors beyond the remodeling of the left atrial could be influencing the increased recurrence of AF post-LAAC. These factors might include the specific effects of the AF ablation and LAAC procedures.

In the case–control study, we demonstrated a significant reduction in the AF-free survival rate in the combined group. Interestingly, the procedure time was not significantly prolonged when LAAC was added following CA in the combined group. Additional analysis found a reduction in the number of additional ablations and ablating attempts in addition to the duration of ablation in the combined group. It implies that insufficient ablation is common following the hybrid procedures of CA and LAAC owing to the intention of reducing the procedural time, which may lead to a recurrence of AF. There might be two reasons for insufficient ablation. One is that we aimed to avoid tissue edema of the ridge, which might contribute to the occurrence of the new peri-device leak associated with the combination strategy [17]. However, it remains controversial as to whether a residual leakage can cause adverse cardiac events. The other reason is to reduce the fluoroscopy time and the use of contrast agents. In this prospective study, we corrected the insufficient ablating strategy and improved the clinical outcome. This rescue study confirmed the adverse effects of insufficient ablation in the hybrid procedure. It is worth mentioning that insufficient ablation was unintentional and should be corrected intentionally.

## 5. Conclusions

We found that insufficient ablation is common in hybrid procedures of CA and LAAC and may lead to the recurrence of atrial fibrillation. It should be corrected intentionally by sufficient ablation of pulmonary vein antrum and additional ablation.

## 6. Limitations

Several limitations existed in this study. First, this study was a single-center clinical trial for Chinese patients, which may introduce selection bias. Second, the prospective study was not a randomized controlled trial. Therefore, the exact impact of insufficient ablation on AF recurrence may be difficult to assess. Third, the symptoms and quality of life of the patients were not assessed in this study, meaning the clinical effect of insufficient ablation was not evaluated comprehensively. Finally, the present study did not include a functional assessment of left atrial reservoir strain by speckle tracking echocardiography. Literature data suggest that an impaired left atrial reservoir strain has been associated with early AF recurrence after electrical cardioversion [24] and after catheter ablation [25, 26].

## Data Availability

The datasets generated and analyzed during this study are not publicly available but are available from the corresponding author on reasonable request. Requests to access the datasets should be directed to Songqun Huang huangsongqun@hotmail.com.

## Supplementary Material

Supplementary material associated with this article can be found, in the online version, at https://doi.org/10.31083/j.rcm2501010.
